# Psychological Aspects of Students With Learning Disabilities in E-Environments: A Mini Review and Future Research Directions

**DOI:** 10.3389/fpsyg.2020.611818

**Published:** 2021-01-07

**Authors:** Stefania Cataudella, Stefano Carta, Maria Lidia Mascia, Carmelo Masala, Donatella Rita Petretto, Maria Pietronilla Penna

**Affiliations:** Department of Pedagogy, Psychology, Philosophy, University of Cagliari, Cagliari, Italy

**Keywords:** e-learning, psychological well-being, emotional distress, self-regulation, learning disabilities

## Abstract

What are the main learning difficulties or advantages encountered by students with learning disabilities (LDs) within e-environments? As a result of the Covid-19 emergency, e-learning is being increasingly used to support students’ learning processes. A number of countries closed their schools altogether, so face-to-face lessons were and have been replaced by distance lessons. A search of current literature via Scopus, Eric and Google Scholar electronic databases was conducted according to Prisma Guidelines. Other sources of literature were also considered, starting from the references in the full text of the articles consulted. We used the following search keywords: “LDs” combined with the “AND/OR” Boolean operator and “e-learning platforms,” “well-being,” “psychological factors,” “emotional distress,” and “self-regulation.” One body of literature highlights the lack of inclusive accessibility standards and a lack of attention to specific tools for addressing LDs, which causes students to develop high levels of stress/anxiety and emotional distress, in addition to low levels of well-being, self-esteem and self-efficacy. Another area of literature looks at how students can develop high levels of self-regulation and emotional awareness, as well as high levels of inclusion. Results are discussed in terms of the promotion of e-learning that focuses on the psychological well-being of students and teachers use of technological tools.

## Introduction

The forced interruption of face-to-face teaching due to the worldwide outbreak of Covid-19, has significantly reactivated the debate on the concrete effectiveness and functionality of e-learning courses. Specifically, our goal was to better understand the psychological effects and efficacy of the current massive use of the e-environments on students with learning disabilities (LDs) (Viner et al., 2020). Literature shows a variety of ways to define e-learning. For example, Cidral et al. (2018) define e-learning as a web-based learning system for the dissemination of information, communication, and knowledge for education and training. Until 2002, Eletti had affirmed that e-learning is a new type of training, a new teaching system that allows you to follow and above all personalize learning. The services and tools used allow for continuous contact with the “student”. In addition, a platform and an interface built *ad hoc*, adapting the contents, allows to model the teaching on the user’s needs (Eletti, 2002). Thus, in light of the massive use of e-environments, there is a definite need to question how effective these tools are for students with LDs. According to international diagnostic criteria, LDs are an overarching group of neurodevelopmental disorders comprising different learning disorders that affect primary and/or secondary academic abilities and a child’s overall capabilities (American Psychiatric Association, 2013; Schulte-Korne, 2014). Children with specific LDs are a rather heterogeneous group, both with regard to specific academic abilities such as listening, thinking, reading, speaking, writing, calculating, and spelling (Sorrenti et al., 2019), as well as to their neuropsychological and functional profiles. For example, they may have impairments affecting different cognitive and neuropsychological abilities (working memory), long-term memory (implicit and explicit memory), attention (selective and sustained), and linguistic, praxis, visuospatial, problem solving, and/or executive abilities (Petretto and Masala, 2017; Visser et al., 2020), etc. Moreover, there is general agreement on the association between LDs and other neurodevelopmental disorders (ADHD and specific language disorders); LDs typically occur in individuals of normal intelligence (Sorrenti et al., 2019). A body of studies indicates a relationship between children’s LD and poor social relations in school (Walker and Nabuzoka, 2007), this aspect is confirmed also in the University context (Filippello et al., 2019). Literature shows a relationship between LDs and internalizing (depressive and anxiety disorders) and externalizing disorders (conduct disorders) (Frith, 2013; Bonifacci et al., 2016; Panicker and Chelliah, 2016; Visser et al., 2020). If LDs are not adequately treated, they can evolve over time, potentially resulting in forms of psycho-social maladjustment (Sorrenti et al., 2019). Regarding the use of e-learning, only a small number of studies have addressed these psychological factors and consequences, and there are few studies which have directly examined the quality of life of students with LDs, or the quality of interpersonal relationships (parents, teachers, and peers). In this mini-review and according to previous research in the field, we analyze these aspects and focus our attention to the following questions:

(1) What are the effects of the use of e-learning on psychological well-being?
(2) What are the effects of accessibility standards in promoting inclusion and in reducing stress, anxiety and emotional distress among students with LDs?

## Methodology

A search of current literature using Scopus, Eric and Google Scholar electronic databases was conducted according to Prisma Guidelines (Moher et al., 2015). Other sources of literature were also considered, starting from the references in the full texts of the articles examined. We used the following search keywords: “LDs” combined with the “AND/OR” Boolean operator and “e-learning platforms,” “well-being,” “psychological factors,” “emotional distress,” and “self-regulation”. Applying a systematic procedure, literature was then selected and results were charted and analyzed. The following inclusion criteria were established: papers on the use of e-learning with LD; on the relationship between e-learning platforms and related psychological aspects (self-esteem, emotional distress, and self-regulation); written in English and published from 2015 to 2020. The following exclusion criteria were applied: systematic reviews; papers on the use of e-learning without LD. On the basis of the research questions and the literature considered, we chose a minireview. For this reason the data will be presented as a narrative review.

## Results and Discussion

In the first part of the search, two independent assessors found 53 articles. Applying our inclusion and exclusion criteria, after reading the abstract, 27 articles were considered. After reading the full texts, 4 further articles were excluded, thus a final group of 23 articles were considered (Table 1). As expected, in literature, regarding the definition of “e-learning”, we found different systems and tools (platforms, devices, web materials/sites, Learning Content Management Systems, ICT, etc.). According to Bjekic et al. (2014) we categorized the different definitions in two groups. The first group refers to the use of Assistive Technology (AT) (hardware or software, used to increase, improve or maintain capabilities of persons with LDs aimed to support and/or increase learning). The second group of e-learning refers to a system of procedures, processes and instructional materials that supports learning. Moreover, we considered a difference between e-platforms and ICT tools (Salehi et al., 2015; Table 2).

**TABLE 1:** Characteristics of papers which met the inclusion criteria.

Author(s), Year	Topic of paper	Country/Countries	Point of view	Accessibility	Methodology	Definition e-learning platforms, instruments and/or devices used	Total no. of subjects (Total no. of subjects with LD)	Type of school/university	Kind of LD	Age range	Assessed Variables
**1.** Adam and Tatnall, 2017	This study investigates whether, and if so how, ICT could be used to support school communities involving students with learning difficulties, and whether it could help these students with their learning in two special School settings	Australia	T/P	No	-Case studies -Interviews with the School Principal, Teachers and Parents	ICT in teaching -ICT was used predominantly to reinforce language and numeracy skills -Classrooms are equipped with an electronic whiteboard and each student has access to a notebook computer and iPad. ICT offered opportunities for students to use technology that would improve their literacy output, access and exposure to technology as well as increasing engagement and provides evidence that scaffolding with a direct teaching approach enhances the learning outcomes of LD students.	-180 students in the first school -About 400 students in the second school (n.s.)	S	n.s.	n.s.	-Attainment in skills and academic knowledge -use of ICT enhances LD students’ independence and equips them with adequate skills which should allow them to continue with further study through various pathways and to move into a normal work environment
**2.** Alamri and Tyler-Wood, 2017	This study investigates which factors associated with learners with disabilities impact student outcomes in an online learning -Environment Successes and struggles in an online setting -Nature of interaction between students with disabilities and instructors	USA-midwest	S	No	Electronic survey of 20 questions	For online courses, the interaction can take place through the use of both synchronous tools (videoconferencing, audio stream, online chat sessions) and asynchronous tools (e-mail, discussion boards).	40 (4LD, 10 ADHD)	U	LD and ADHD	18–58	-Social presence, -interpersonal relationships -Achievement -Satisfaction -different types of interactions in online learning: learner–interface interaction, learner–content interaction, and learner–learner interaction.
**3.** Baharuddin and Dalle, 2019	The study uses a four-phase iterative process to develop and analyze a prototype eLearning system: understanding the problem, designing the system, developing the system, and gathering user feedback	Indonesia	T/S	No	Interviewed observation during the use of ICT	Computer connected to the Internet, virtual classrooms	17 teachers (n.s.)	S	n.s.	n.s.	Communication Attendance Reasonable accommodations Knowledge and competencies of teachers
**4.** Benmarrakchi et al., 2017	This study investigates the potential benefits offered by the use of Information and Communication Technology (ICT) to support dyslexic students by considering their preferred learning styles. Based on the results of the analysis of learning styles differences, the authors introduced an adaptive mobile learning to support and promote learning for dyslexic students.	Morocco	S	No	Questionnaires	ICT (digital technologies Multimedia applications) adaptive mobile learning	28 (8)	PS	dyslexia	8–10	Learning styles (description of the attitudes and behaviors, which determine an individual’s preferred way of learning VAK learning style model is based on three main sensory receivers: visual (V), auditory (A), and kinesthetic (K). - ‘cycle of learning’, four-stage cycle of learning: abstract conceptualization (AC), concrete experience (CE), active experimentation (AE), and reflective observation (RO).
**5.** Berizzi et al., 2017	This study investigates the effect of an attributive-metacognitive training on attributional style of students with Special Educational Needs (SEN), proposed by a elearning platform	Italy	S	Yes	Questionnaires	ICT (e-mail, Skype network conversations, videoconferencing, e-learning platforms, such as Moodle, Edmodo, and others)	30 students with special educational needs (30)	SS	24 dyslexia 6 ADHD	11–15	Metacognition Self esteem Self efficacy Locus of control
**6.** Chen et al., 2015	This study aims to explore the learning experience of learners with dyslexia when reading passages using different online reading affordances to derive some guidelines for dyslexia-friendly online text.	Malaysia	S	Yes Web site accessibility guidelines and role of engagement	Qualitative multiple-case study	Web sites The use of online learning is appropriate for learners with dyslexia as this delivery mode allows self-paced learning and affords multimodal technologies that have the potential to settle dominant deficit models of dyslexia	12 (12)	SS	Dyslexia	14–18	Online reading affordances Perceived learning behavioral engagement (BE), cognitive engagement (CE) as well as affective engagement (AE) web accessibility guidelines for users with dyslexia
**7.** García-González et al., 2020	This study investigates how students perceived access to higher education -Role of barriers	Spanish	S	Yes	Focus group and interviews Discourse analysis	Personal Learning Environments PLE WEB	16 (3)	U	Dyslexia	20–29	Web or computer barriers, Learning barriers, burocratic barriers, architectural barriers, social barriers
**8.** Kent et al., 2018	This study investigates both the attitudes of students with disability towards disclosure of their disabilities and their experiences ofstudying online and accessibility of online learning materials	Australia	S	Yes Accessibility on web materials Need for a more interest toward Universal Design	Online survey	E-learning in open university	2000 students with disability (16,3% with LD)	U		Mean age 36	Accomodations and disclosure of disabilities and the difficulties for students to disclosure their disabilities, even if this makes difficult to personalized accomodations.
**9.** Lambert and Dryer, 2018	This study investigates the effects of learning challenges on online learning environments on the quality of life of students with learning disabilities	Australia	S	Yes	Semi-structured interview	Online learning environments	8 (8)	U	Dyslexia	21–43	Stress, quality of life, anxiety, self esteem
**10.** Lipka et al., 2019	This study investigated how students and instructors perceived the instruction in adapted courses in post secondary school students	Israel	S/T	No	Semi-structuredinterviews	Adapted courses	1000 (5)	U	LD and ADHD	20	Perception of teaching Locus of control emotional support
**11.** Naumova et al., 2017	This study investigates an integrated educational methods of training matching the features of disabled students. The technology includes both traditional and innovative methods of training	Russia	S	Yes	n.s.	Adapted courses with online web content. Information technologies Web content has to be available for a wide range of users with health limitations.	6 (n.s.)	U	n.s.	n.s.	Educational Motivation Interpersonal Relationship
**12.** Nieto-Márquez et al., 2020	This study investigates effects of digital teaching on metacognitive skills	Spain	S	No	Questionnaire	Digital teaching platform called Smile and Learn,	130 (n.s.)	PS	n.s.	8	Metacognitive skills
**13.** Ouherrou et al., 2019	This study explored the benefits of ICT use to identify the ways in which emotions are involved during the learning process in Virtual Learning Environments (VLE)	Morocco	S	Yes		ICT Virtual learning environment Artificial intelligence Educational games	42 (14)	PS	LD	7–11	Affective state Emotions Facial expression recognition
**14.** Rice and Carter, 2016	This study investigates how practicing teachers provided self-regulation strategies to students with disabilities in a fully online learning environment. In this context, the teachers intended to offer self-regulation strategies to students, but they were largely unable to do so.	Kansas	T	Yes		E-learning environment	Teachers (n.s.)	S	n.s.	n.s.	Emotional demands Selfregulation In students with disability
**15.** Richardson, 2015	This investigation studied attainment in students with dyslexia or other specific learning difficulties who were taking modules by distance learning with the Open University in 2012. Students with dyslexia or other specific learning difficulties who had no additional disabilities were just as likely as nondisabled students to complete their modules, but they were less likely to pass the modules that they had completed and less likely to obtain good grades on the modules that they had passed.	UK	S	Yes		Distance learning in open university computer-based support, particularly CD-ROMs, dedicated websites and computer-mediated conferencing	4961 (n.s.)	U	Dyslexia	21–60	attainment
**16.** Richardson, 2016	This study examined the experiences of students taking the same courses in the humanities by distance learning when tutorial support was provided conventionally (using limited face-to-face sessions with some contact by telephone and email) or online (using a combination of computer-mediated conferencing and email).	UK	S	yes		In distance learning, the curriculum was traditionally provided through correspondence materials. Nevertheless, most distance-learning institutions use various kinds of personal support in trying to narrow what Moore (1980) called the “transactional distance” with their students, most commonly through regular albeit limited tutorials. In recent years, there has been an increasing use of information technology in distance education, with a move from paper-based to electronic materials accompanied by a move from face-to-face to online tutorial support	292 (24)	U	Dyslexia	n.s.	The results showed that, given a choice between face-to-face and online tutorial support, students with and without disabilities were equally likely to choose online support rather than face-to-face support
**17.** Sharabi et al., 2016	This study investigates personal resources, loneliness, and academic self-efficacy among college students with and without LD in smartphone and internet use	Israel	S	No	Questionnaire	Smart phones Internet	178 (59)	Transition to college	n.s.	24 mean age	Coping, Self-efficacy Predictors of loneliness Hope, optimism Sense of coherence
**18.** Shonfeld and Ronen, 2015	This study investigates how adapting the online course by using information and communication technology following formative assessment will improve students’ self-learning ability as well as broaden their science knowledge, their lab performance and teaching skills. The study was focused on preparing K-2 pre-service teachers	n.s.	S	No	Questionnaires and interviews	Online learning as a teaching tool, the challenge of adapting a course for three groups of students: students with learning disabilities, excellent students, and average students	121 (25)	U	n.s.	n.s.	The online course was based on the Highlearn platform which enabled ICT learning synchronously through InterWise. The course included peer teaching: students conducted group discussion and peer feedback; individual monitored learning. All students were instructed by the lecturer in developing
**19.** Smith et al., 2016	This study investigated parent perceptions and experiences regarding fully online learning for their children with disabilities	Kansas	P	Yes	Interviews	Online learning in online schools	18 (Parents of children) (7)	S	Learning disabilities	n.s.	Parental role on online learning Communication
**20.** Straub and Vasquez, 2015	This study investigates online writing instruction for students with learning disabilities (LDs) using synchronous online collaborative writing software to investigate effects of self-regulated strategy development for strategy instruction in persuasive writing.	USA Florida	S	No	n.s.	Writing Instruction Online	4 (4)	S	n.s.	adolescent	Self-regulated strategies development
**21.** Terras et al., 2015	This study investigated how online learning may afford students with disabilities enhanced opportunities for academic success. In this study, the authors interviewed 11 graduate students to determine their experiences with disability accommodations in online courses and their perceptions of the relationship between those accommodations and their academic success.	North Dakota	S	Yes	n.s.	Accommodation in online courses Since students with disabilities may have difficulty concentrating, staying on task, and adhering to a schedule, online settings (particularly those that are asynchronous) allow students to access courses anywhere, anytime, and any place and provide “the personalized time they need to think, process, and respond”.	11 (4 LD 2 ADHD)	U	Learning disabilities ADHD	22–55	Disability accommodations in online courses students responsibility instructor responsibility University responsibility
**22.** Vasalou et al., 2017	This study investigates the case of a digital game called Words Matter. The game was designed for children with dyslexia and was informed by principles from casual games and evidence-based practice from special education. Focusing on the game play of two groups of children, we employ a systematic thematic analytic approach on videos of children’s verbal and non-verbal interaction triangulated with their game logs, concentrating on the nature of student-student as well as student-tutor social interactions.	UK	S	No	Case studies	Drill and practice digital games-based learning Games-based pedagogies for students with special education needs	8 (8)	S	Dyslexia	11–12	Motivation Engagement on learning Social engagement Self-esteem Personal identity Peer tutoring
**23.** Ziadat, 2019	This study investigates the impact of e-Learning on the development of academic and social interaction skills among students with learning disabilities in Jordan from the perspective of their teachers	Jordan	T	No	n.s.	Multimedia and information technologies; as well as the use of the internet as a new technique of teaching, The internet has become one of the most important ways to make available resources and to share and acquire information. No common definition of the term e-learning.	100 teachers (n.s.)	S	n.s.	n.s.	Role of e-learning on the development of academic skills among students with learning disabilities Social interaction Social behaviour

***Point of view:** Students (S), Parents (P), Teachers (T); **Type of school/university:** Primary School (PS), Secondary School (SS), High School (HS), University (U)**;** not specified: n.s.; Special educational Needs/Special Education Needs: SEN. **Accessibility:** Yes or No (considered/not considered in the article).*

**TABLE 2:** Papers which met the inclusion criteria in the school setting analyzed according to Bjekic et al. (2014).

School	E-learning (N Studies)		Limitations	Strenghts
	ICT	E-platforms		
**Primary/Secondary School**	4(3*)	8**(6*)	− Lack of interest:	− Promotion of:
			• technology in the development of	• skills and academic knowledge
			student curriculum	educational outcome
			design framework for digital materials	students’independence and self-regulation
			personalized paths parent’s training in supporting children’s e-learning experience	pathway for the transition from school to further study learning styles
				communication among children, teachers and parents
				metacognitive experience
				emotional well-being
				tutor/teacher scaffolding
				parents support
**High school**	1*	1*	− Lack of:	− Promotion of:
			• design interface	• tutor/teacher scaffolding
			appropriate online instruction strategies	social support
				stress and anxiety reduction
**University**		12 (3*)	− Lack of:	− Promotion of:
			• longitudinal studies	• scaffolding (instructor-learner interaction)
			self-regulation and emotional well-being	social support
				metacognitive interventions
				academic retention

*^∗^Presence of Assistive Technology. ^∗∗^Three studies with E-platforms and ICT.*

The papers showed a certain amount of heterogeneity in their definition of LDs. Some authors proposed a specific definition (Chen et al., 2015; Richardson, 2015; Shonfeld and Ronen, 2015; Straub and Vasquez, 2015; Benmarrakchi et al., 2017; Sharabi et al., 2016; Adam and Tatnall, 2017; Vasalou et al., 2017; Lambert and Dryer, 2018; Lipka et al., 2019; Ziadat, 2019), while others proposed a general reference to Special Educational Needs or used the World Health Organization definition of Disability (World Health Organization, 2001; Berizzi et al., 2017; Naumova et al., 2017; García-González et al., 2020). Some papers reported the definition of LD based on international diagnostic criteria, others described specific national law/s or references (Sharabi et al., 2016). Moreover, with regard to sample recruitment, some authors chose samples consisting of different groups of students with other kinds of disabilities and then specified the number of students with LDs (Richardson, 2015, 2016; Shonfeld and Ronen, 2015; Terras et al., 2015; Benmarrakchi et al., 2017; Sharabi et al., 2016; Alamri and Tyler-Wood, 2017; Berizzi et al., 2017; Kent et al., 2018; Lipka et al., 2019; Ouherrou et al., 2019; García-González et al., 2020); while in other papers, the sample is made up only of students with LDs (Chen et al., 2015; Straub and Vasquez, 2015; Vasalou et al., 2017; Lambert and Dryer, 2018). Regarding the level of schooling, about 1/2 of the studies focused on University environments (Richardson, 2015, 2016; Terras et al., 2015; Alamri and Tyler-Wood, 2017; Naumova et al., 2017; Kent et al., 2018; García-González et al., 2020) and the other 1/2 examined primary and secondary schools (Chen et al., 2015; Straub and Vasquez, 2015; Benmarrakchi et al., 2017; Rice and Carter, 2016; Smith et al., 2016; Adam and Tatnall, 2017; Berizzi et al., 2017; Vasalou et al., 2017; Baharuddin and Dalle, 2019; Lipka et al., 2019; Ouherrou et al., 2019; Ziadat, 2019; Nieto-Márquez et al., 2020). One paper focused on the transition from school to university (Sharabi et al., 2016). As expected, we also found a considerable heterogeneity in school settings, ranging from mainstream school/classrooms to special needs schools/classrooms, according to specific national and theoretical approaches and policies regarding the field of inclusion (see Table 2). Given that the countries in our sample ranged across Europe, United States, as well as Arab and Slavic countries, there was some diversity in the idea of inclusive policies for students with LDs. This is due to national differences regarding the issues of policies for students with LDs and, in general, for students with SEN. In some countries, there is an inclusion-based approach where students with LDs are placed in mainstream schools; in other countries there are special schools and special classrooms for them. In some countries, transition to complete inclusion is still ongoing (Lindsay, 2016; Norwich, 2016; Petretto et al., 2019; Pilia, 2019). While one of the papers described a specific experience in two special needs classes (Adam and Tatnall, 2017), other research papers concentrated on the use of specific e-learning approaches to designated groups of children with LDs or to all the children in the classroom in mainstream schools (Straub and Vasquez, 2015; Vasalou et al., 2017).

The approaches employed range from the use of specific devices and/or platforms, to the use of specific “reasonable accommodations” (such as font quality and sizes in the learning materials on the web or the use of specific support technologies) (Chen et al., 2015; Benmarrakchi et al., 2017; Rice and Carter, 2016; Alamri and Tyler-Wood, 2017; Berizzi et al., 2017; Ouherrou et al., 2019; García-González et al., 2020); or the use of software/games aimed to increase specific abilities in students with LDs (Straub and Vasquez, 2015; Vasalou et al., 2017). For university settings, some articles describe the experiences of so-called “Open universities” that have been based on distance learning methods since they started. With the development of ICTs, in the past few decades these universities have started to use e-learning platforms to contact students and to promote learning and social connections (Richardson, 2015, 2016; Kent et al., 2018). Their ongoing experiences focus mainly on the attainment of students with LDs as well as on the need to increase access to information and learning. Other studies focus on the need for dedicated online courses to specific categories of students, aiming at reducing barriers and distances and providing specific accommodations (Terras et al., 2015).

The age range in these university samples is very wide. From a positive perspective it can represent a sign of the wider opportunity for older people to access university courses. However, according to some studies, it could be also the sign of a lower and slower attainment of students with LDs in University (Richardson, 2015, 2016; Shonfeld and Ronen, 2015). The topics of attainment and achievement are interesting because even though some papers have discussed the risk of low achievement for students with LDs, other studies have demonstrated the positive effect of accommodations and have showed examples of unexpected achievement by LD students (Shonfeld and Ronen, 2015). Another aspect is the fear of disclosure of their diagnosis by some students with LDs and the effects on their tendency to hide diagnoses rather than to communicate it, even when they should do so in order to define specific “reasonable accommodations” (Richardson, 2015, 2016; Terras et al., 2015). Although there may be increased student awareness of the need to disclose their diagnosis and the functional profiles that help to define a personalized approach that facilitates their access to learning and materials, some authors have highlighted the importance of further discussing the role of communication between teachers/instructors and students with LDs in the development of more comfortable learning environments and in the pursuit of shared learning and achievement aims (Terras et al., 2015).

### Focus on Psychological Well-Being

Few studies have directly examined the psychological aspects of students with LDs in e-environments. Some papers have focused on psychological consequences of the intensified use of Information and Communications Technologies (ICTs); other papers instead focused especially on adults, addressing some psychological effects of e-learning procedures adapted to students with LDs. In their study, Ouherrou et al. (2019) highlighted the fact that the integration of ICTs in special needs education may have a positive impact on the emotional states of children with LDs, because they may experience fewer negative emotions than findings of current literature would suggest with regard to the presence of higher levels of negative emotions in the classroom. Vasalou et al. (2017) argued that a socially constructed view of digital games-based learning provides new opportunities for the support of children with dyslexia. Children spontaneously engage in “game talk” regarding game performance, content, actions and they strategically use their individual game experiences to express their personality and interact with their peers. Also, such experiences can help improve the intra-individual function by enhancing a child’s self-esteem. The findings of Sharabi et al. (2016) supported earlier studies that assessed children and adolescents with LDs (Sharabi and Margalit, 2014), showing that college students with LDs possess lower levels of personal resources (sense of coherence, hope and academic self-efficacy) and suffer higher levels of social distress and loneliness than their peers. The loneliness factor was predicted by measuring online avoidance coping, their amount of smartphone use and by examining their personal resources, the use of ICTs may provide additional environmental conditions to enable youngsters to meet their emotional needs. At the same time, these opportunities may also be misused as avoidance coping and thus may contribute to increased loneliness and lower academic self-efficacy. Coherently with previous studies, Lambert and Dryer (2018) highlighted that in high education the e-environment had a negative influence on the quality of life of students with increased stress and anxiety, the perception of feelings of inadequacy, a decrease in time available for other activities and personal relationships. The same authors also highlighted that for many students, the academic and emotional support provided by family and friends was a key factor in study success. Studies on the perception of the impact of e-learning on the development of academic skills and social interaction from the perspective of students and/or teachers showed that the quality of teacher-student relationships contribute to producing improvements in learning achievement (Alamri and Tyler-Wood, 2017; Lipka et al., 2019; Ziadat, 2019). Only a small number of studies have considered the role of parents. Smith et al. (2016) investigated parents’ perceptions and experiences regarding exclusive online learning for their children with disabilities. The results showed that this experience altered parents’ previous roles and that many parents were not equipped to take a teaching role due to lack of training, time, and other constraints. A parent-as-teacher role can negatively affect parent–child dynamics, leading to frustration for parent and child but full online learning requires increased parent–teacher communication. This increased level of interaction and the positive outcomes associated with the shared information enhanced a collaborative parent–teacher relationship. The use of ICT and e-learning can improve the learning of students with LDs only where a supportive context is present. The support provided by family, teachers and peers can create a protective factor which improves the well-being of students with LDs.

### Focus on the Accessibility Standards and Emotional Distress

Many of the difficulties in designing e-learning courses are due to accessibility issues that can affect successful engagement (Draffan, 2012; Seale, 2013). The heterogeneity of the LD population entails great challenges to all parties involved in creating, managing and using e-learning content, tools and platforms with accessibility features (Guenaga et al., 2004; Baharuddin and Dalle, 2019). Some papers described the risks of a design approach based on a general and average idea of students without LDs (Kent et al., 2018). For Beacham and Alty (2006) the e-learning materials commonly employed were developed with the needs and capabilities of non-dyslexic learners in mind; clearly, resources do not generally take into consideration the individual learning approaches that these students manifest (Alsobhi and Abeysinghe, 2013; Chen et al., 2015; Luongo, 2018). Chen et al. (2015) also underline this point, observing that empirically derived guidelines for designing accessible online learning environments for learners with dyslexia are still scarce. The problem of accessibility is fundamental in e-learning design, as it is strictly linked to certain psychological factors that will affect students, like willingness to focus on learning, management of emotions and behavior, learning motivation, interest and self-regulation (Chen et al., 2015; Berizzi et al., 2017; Luongo, 2018). Existing literature provides clear evidence that text-based synchronous activities commonly used in education, like chat programs and videoconference, can create psychological and learning difficulties. However, only a small number of papers take into account the problems of students with LDs in collaborative environments (Luongo, 2018). Some papers focus on the positive aspects of the use of e-learning platforms in increasing accessibility to information and learning materials (Richardson, 2016), above all because participation in remote activities, like on-line forum discussions, improves the autonomy and self-regulation of students (Berizzi et al., 2017). These aspects are reinforced by continuous support of tutors and peers, and reflection on what has been done, the goals to be achieved, and ultimately the strategies to be adopted. Other articles described the possible role of a “universal design for learning approach” in the design of websites, web materials and e-learning platforms (Chen et al., 2015; Shonfeld and Ronen, 2015; Alamri and Tyler-Wood, 2017; Kent et al., 2018; Nieto-Márquez et al., 2020) in order to create environments that can be useful also for students with LDs.

## Conclusion

This mini-review has attempted to analyze both the quality of life of students with LDs and their interpersonal relationships and the features of e-learning that can have positive and negative effects on them. The considerable heterogeneity of the articles we selected led us to the following reflections: we are aware that the heterogeneity could represent a limit but also an expected consequence of the chosen way of to explore a complex topic. Bearing in mind this issue, in a following article we will discuss the picture of the state of art that we derived from this minireview. In the near future, we will explore specific and more focused aspects, also with an attention on intervention aims. Two issues are emerged.

The first is how important online-support is to consolidate teacher-learner relationships, as it can affect a student’s well-being and learning achievement. We know that e-learning is a psychological process supported by e-technology, and learning is a social activity. Understanding that it is socially constructed should ensure that e-learning is organized to promote participation, allowing all students to take part in all activities, thus enhancing cooperative-learning.

The second consideration regards the fundamental role of accessibility and “reasonable accommodations”, which should lead to a reduction of emotional distress and promote positive psychological factors through full engagement with e-learning. In order to be effective, e-learning must go beyond simply digitizing books and ought to be designed carefully and appropriately for learners (Penna and Stara, 2007, 2010). What about the current and ongoing experience of the massive use of e-learning due to the COVID-19 outbreak? We agree with Al Lily et al. (2020), who coined the term “Crisis Distance learning,” that the current ongoing experience is different from previous ones, and that caution is needed before making any kind of generalizations from previous experiences. Nevertheless, some general considerations can be drawn for future research. It is necessary to encourage and maintain cooperative approaches in all spheres, including in the use of e-learning in school and universities, with particular attention on the quality of the relationships between all the people involved (students-teachers-parents-peers) and with an even more specific focus on the psychological needs of students with LDs. The improvement of e-learning systems designed with attention to the care and quality of relationships can promote well-being among all parties involved in the learning process.

## Author Contributions

All authors equally contributed to the design of the study. All authors have read and agreed to the published version of the manuscript.

## Conflict of Interest

The authors declare that the research was conducted in the absence of any commercial or financial relationships that could be construed as a potential conflict of interest.
